# Development of an Anthropomorphic Phantom of the Axillary Region for Microwave Imaging Assessment

**DOI:** 10.3390/s20174968

**Published:** 2020-09-02

**Authors:** Matteo Savazzi, Soroush Abedi, Niko Ištuk, Nadine Joachimowicz, Hélène Roussel, Emily Porter, Martin O’Halloran, Jorge R. Costa, Carlos A. Fernandes, João M. Felício, Raquel C. Conceição

**Affiliations:** 1 Instituto de Biofísica e Engenharia Biomédica (IBEB), Faculdade de Ciências, Universidade de Lisboa, Campo Grande, 1749-016-Lisbon, Portugal; rcconceicao@fc.ul.pt; 2 Sorbonne Université, CNRS, Laboratoire de Génie Electrique et Electronique de Paris, 75252, Paris, France; soroush.abedi@sorbonne-universite.fr (S.A.); joachimowicz@paris7.jussieu.fr (N.J.); helene.roussel@sorbonne-universite.fr (H.R.); 3 Université Paris-Saclay, CentraleSupélec, CNRS, Laboratoire de Génie Electrique et Electronique de Paris, 91192, Gif-sur-Yvette, France; 4 Translational Medical Device Lab, National University of Ireland Galway, H91 ERW1 Galway, Ireland; niko.istuk@nuigalway.ie (N.I.); emily.e.porter@ieee.org (E.P.); martin.ohalloran@gmail.com (M.O.H.); 5 Department of Electrical and Computer Engineering, The University of Texas at Austin, Austin, TX 78712, USA; 6 Instituto de Telecomunicações, Instituto Superior Técnico (IST), Universidade de Lisboa, 1049-001 Lisbon, Portugal; jorge.costa@iscte-iul.pt (J.R.C.); carlos.fernandes@lx.it.pt (C.A.F.); joao.felicio@lx.it.pt (J.M.F.); 7 Departamento de Ciências e Tecnologias da Informação, Instituto Universitário de Lisboa (ISCTE-IUL), 2649-026 Lisbon, Portugal; 8 Centro de Investigação Naval (CINAV), Escola Naval, 2810-001 Almada, Portugal

**Keywords:** anthropomorphic phantom, axillary lymph node diagnosis, biological tissue dielectric properties, breast cancer, microwave imaging, open-ended coaxial-probe, pre-clinical testing

## Abstract

We produced an anatomically and dielectrically realistic phantom of the axillary region to enable the experimental assessment of Axillary Lymph Node (ALN) imaging using microwave imaging technology. We segmented a thoracic Computed Tomography (CT) scan and created a computer-aided designed file containing the anatomical configuration of the axillary region. The phantom comprises five 3D-printed parts representing the main tissues of interest of the axillary region for the purpose of microwave imaging: fat, muscle, bone, ALNs, and lung. The phantom allows the experimental assessment of multiple anatomical configurations, by including ALNs of different size, shape, and number in several locations. Except for the bone mimicking organ, which is made of solid conductive polymer, we 3D-printed cavities to represent the fat, muscle, ALN, and lung and filled them with appropriate tissue-mimicking liquids. Existing studies about complex permittivity of ALNs have reported limitations. To address these, we measured the complex permittivity of both human and animal lymph nodes using the standard open-ended coaxial-probe technique, over the 0.5 GHz–8.5 GHz frequency band, thus extending current knowledge on dielectric properties of ALNs. Lastly, we numerically evaluated the effect of the polymer which constitutes the cavities of the phantom and compared it to the realistic axillary region. The results showed a maximum difference of 7 dB at 4 GHz in the electric field magnitude coupled to the tissues and a maximum of 10 dB difference in the ALN response. Our results showed that the phantom is a good representation of the axillary region and a viable tool for pre-clinical assessment of microwave imaging technology.

## 1. Introduction

Breast cancer is the most frequently diagnosed and cause of cancer death among women [1]. Metastases (i.e., cancer’s spread to secondary locations) are the leading cause of death for patients suffering from breast cancer. Seventy-five percent of the lymph from the breast drains into Axillary Lymph Nodes (ALNs), making them the first location where breast metastases migrate. ALN diagnosis is essential in breast cancer as the disease status of these nodes is important to determine the staging of the pathology before therapeutical decision [2]. The state-of-the-art method for ALN diagnosis is Sentinel Lymph Node Biopsy (SLNB), which consists of the surgical excision and histological examination of the first regional node (or nodes) to drain the primary tumor. However, in the clinical practice, the sentinel ALN positive diagnosis often leads to the pre-emptive removal of all the regional ALNs, causing longer recovery of patients, risk of infection and lymphedema [3,4]. Standard imaging modalities, such as Magnetic Resonance Imaging (MRI) or the combination of Positron Emission Tomography and Computed Tomography (PET-CT), present an alternative, but their use is associated with high costs and, in the case of PET-CT, to radiation exposure. Thus, there is a clinical need for an alternative technology which can diagnose ALNs non-invasively.

Microwave Imaging (MWI) is an emerging technology, which is being proposed for several applications, including breast cancer screening [5,6,7]. This technique relies on the contrast of dielectric properties between healthy and malignant tissues at microwave frequencies (typically in the 1 GHz–10 GHz range). MWI presents several advantages compared to other imaging systems used for breast cancer screening, namely it is low-cost, non-invasive, portable, and it employs non-ionizing radiation. A MWI system dedicated to ALN diagnosis is under study in our research group [8,9,10,11], and, in recent years, other authors addressed the possibility of imaging ALNs with this technique [12]. A critical step to assess the viability of this technology is to test it by means of numerical simulations and experiments on anthropomorphic phantoms. Numerical and physical anthropomorphic phantoms allow researchers to assess the system performance in a controlled and realistic environment.

For proper evaluation of MWI technology, the employed phantom should (i) be representative of the body anatomy, in terms of morphology and tissues considered, and (ii) accurately mimic the dielectric properties of the tissues of interest. Additionally, it is useful if the phantom is (iii) re-configurable, so that organ dielectric properties, shape, and position can be adjusted to mimic the physiological variability of tissue dielectric properties or morphology of individuals. Lastly, it is useful if the shape and the dielectric properties of the physical phantom are (iv) stable over time, in order to enable repeatable measurements.

Several authors proposed various alternatives to model anatomical regions for MWI applications. Examples include breast [13,14,15,16,17,18,19,20,21,22,23,24], and head phantoms [24,25,26]. However, outside our research groups, no one has yet developed an antropomorphic phantom of the axillary region suitable for MWI evaluation.

Here, we created an anatomically and dielectrically realistic phantom representative of the female axillary region for the assessment of MWI of the ALNs. To this end, we segmented a thoracic CT scan and we identified five tissues of interest for realistic representation of the underarm region, given the intended application: fat, muscle, bone, lung, and ALNs.

However, there is a gap in knowledge regarding the dielectric properties of ALNs which prevents the realization of such a phantom. In fact, only a few studies reported the dielectric properties of ALNs in the literature. Choi et al. [27] measured 27 ALNs from 12 patients diagnosed with invasive breast carcinoma. The study was conducted using the Open-Ended Coaxial-Probe (OECP) measurement method and data were sampled over the range from 0.5 to 30 GHz. The authors concluded that ALNs dielectric properties (average relative permittivity around 10 to 15 for healthy ALNs, and around 35 to 40 for metastasized ALNs, at 4 GHz) are sensitive to cancer cell presence. However, the heterogeneity of samples was not discussed. This can be critical if we consider that, after their excision, ALNs are often surrounded by a variable amount of fat, which can affect the measured permittivity. Cameron et al. [28] reported 30 OECP measurements of 23 ALNs, excised from 14 patients, in the frequency range from 0.5 to 20 GHz. Measurements were performed immediately after node excision, during breast cancer surgeries. For all the measurements, the probe was placed in contact with the external surface of the node; while for two of the analyzed ALNs, measurements were completed when placing the probe directly on the cross-section of the ALN (after slicing it). The results showed very high variance in dielectric properties (permittivity approximately ranging from 5 to 55 at 4 GHz). The authors concluded that fatty tissue tended to dominate the measurements when placing the probe in contact with the exterior of the node and assumed that measurements with relative permittivity in the range of 1 to 10 corresponded to samples that were predominantly fat. For those two ALNs which were sliced in half, the interior of the node was measured. The two inner cross-section measurements resulted in higher dielectric properties with respect to those conducted on the corresponding outer surfaces (covered by fat), suggesting that measurements of the interior of the ALN may be more representative of the actual ALN dielectric properties. Despite concluding that fat presence on the outer surface highly influences the measurements, that study is limited to the full analysis of dielectric properties of only two ALNs.

Given the limitations on ALN dielectric studies found in the literature, we characterized the complex permittivity of LNs in the frequency band of 0.5 GHz to 8.5 GHz using the OECP method. We measured human ALNs excised from patients who underwent SLNB during breast cancer surgery. However, ALN sample handling constraints prevented us from measuring their inner content. Hence, to overcome the sample handling restrictions, we measured animal LNs, since we were able to assess their inner content.

We fabricated tissue mimicking materials (TMMs) using the dielectric properties found in the literature [29,30] or measured in the previous task. We fabricated liquid TMMs, which filled 3D-printed hollow containers representing the tissues of interest.

Lastly, we quantified—through full-wave simulations—the differences between our phantom and the real axillary region, namely the influence of the plastic containers on the electric field (E-field) coupled to the phantom. This is an important validation to confirm the phantom is representative of a real scenario. To the best of our knowledge, this is the first realistic phantom of the axillary region for MWI experimental assessment.

The paper is organized as follows: Section 2 reports the complex permittivity of LNs measured from animal LNs and human ALNs using the OECP technique; Section 3 describes the fabrication and the dielectric characterization of the TMMs; Section 4 describes the development of the numerical model of the axillary region and the fabrication of the 3D-printed phantom; Section 5 discusses the representativeness of the fabricated phantom compared to the actual axillary region, namely the influence of polymer presence on the energy coupling to the axillary region; Section 6 draws the main conclusions and presents future work perspectives.

## 2. Dielectric Properties Measurement of Lymph Nodes

In the development of an anthropomorphic and dielectrically realistic phantom for MWI applications, it is essential to have knowledge of the dielectric properties of the tissues to be mimicked. However, data reporting on ALN permittivity is rather limited. Therefore, we characterized human and animal LNs at microwave frequencies. We firstly performed dielectric measurement of human ALNs (Section 2.1.1). However, due to tissue handling restrictions, the measurements were limited to the outer surface of the ALNs, allowing limited conclusions to be drawn. Consequently (Section 2.1.2), we extended the study to animal LNs, which were sliced in order to enable the measurement of their inner cross-section. Animal LN measurements helped us interpret the measurements performed on human ALNs. We would like to note that most of the tissue dielectric properties available in the literature [29] are the result of ex-vivo animal tissue dielectric measurements. We also note that animal and human LNs have the same anatomy and physiological functioning.

Prior to the presentation and discussion of the results, it is useful to recall the fundamentals of material dielectric properties at microwave frequencies. Any dielectric material is characterized by its relative complex permittivity, $\epsilon_{c}$, and relative complex permeability, $\mu_{c}$. The latter is assumed to be $\mu_{c}=1$, as all the materials involved in this work are non-magnetic. As for $\epsilon_{c}$ it is defined by its real, $\epsilon^{\prime }$, and imaginary parts, $\epsilon^{\prime \prime }$, as follows:

(1) $$\epsilon_{c}\left(f\right)=\epsilon^{\prime }\left(f\right)-j\epsilon^{\prime \prime }\left(f\right).$$

The real part, also referred to as dielectric constant, denotes the ability of the material to store energy in response to an applied E-field, whereas the imaginary part accounts for the losses in the material. The latter can be used to define an equivalent conductivity $\sigma \left(f\right)=\omega \epsilon^{\prime \prime }$, where ω=2πf, in which *f* is the frequency. Note that $\epsilon_{c}$ may exhibit dispersive behaviour, as it is a function of frequency. The dispersion of biological tissue permittivity is commonly represented in literature in the parametric form defined by the n-pole Debye model as follows:

(2) $$\epsilon \left(\omega \right)=\epsilon_{\infty }+\sum_{i=1}^{n}\frac{\Delta \epsilon_{i}}{1+j\omega \tau_{i}}-\frac{\sigma_{s}}{j\omega \epsilon_{0}},$$

where $\epsilon_{\infty }$ is the infinite limit of the permittivity, Δϵ is the change in permittivity, $\sigma_{s}$ is the static ionic conductivity, $\tau_{i}$ is the relaxation constant, and n is the number of poles.

### 2.1. Measurement Procedure

**Figure 1:**
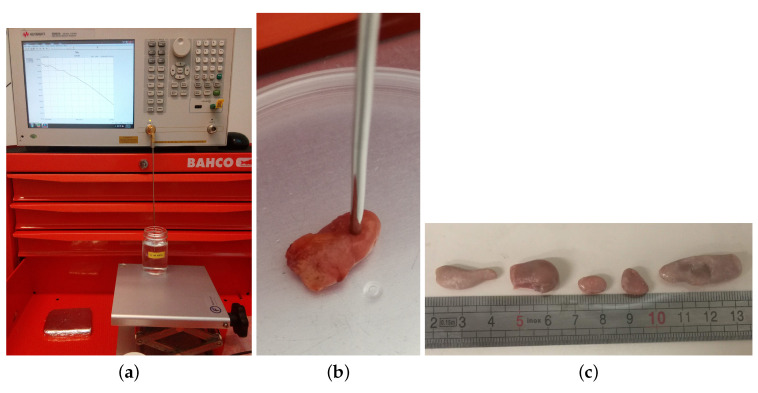
(**a**) Dielectric measurement setup: the open-ended coaxial-probe is connected with a right angle SMA-connector to a vector network analyzer during the measurement of the validation load (0.1 Molar NaCl water solution). (**b**) Detail of a human axillary lymph node measurement. (**c**) Five sheep inguinal lymph nodes that have been measured.

The dielectric measurements were performed using the Keysight slim form probe connected to the Keysight E5063A Vector Network Analyser (VNA) through a right angle SMA-connector. With this procedure, we avoided the use of cables which could introduce a potential source of uncertainty [31]. The measurements covered the frequency bandwidth of 500 MHz–8.5 GHz. Prior to measurements, the system was calibrated using the open/short/load method, as is standard procedure [32]. We used deionized water as a standard load. A picture of the measurement setup is reported in Figure 1a.

The OECP measurement technique is very sensitive to VNA drift, as well as inappropriate handling of the probe [31,33]. As a result, we performed an additional measurement to validate the calibration. This measurement was done with a 0.1 Molar Sodium Chloride (0.1M NaCl) solution, in which complex permittivity is well documented in Reference [34] over frequency and temperature, in order to estimate the error of the measurement setup at any given time during a measurement campaign. This validation procedure was firstly proposed in Reference [31], and it is currently accepted by the scientific community to estimate the measurement error and to minimize the uncertainty inherent to the measurements. In the present study, we performed the validation measurement immediately after setup calibration (prior to tissue measurements) and after some sample measurements (usually within 90 min after the calibration). We recorded the temperature of the validation solution so that we could match it to the correct model. When the error was above 10%, the probe calibration and validation were repeated. Table 1 presents the average percentage error (over frequency and over all measurement sessions) of the validation measurements. We ensured the error was kept below 8%, which is acceptable for the purpose of tissue characterization.

Regarding tissue measurement, we are aware that the pressure applied with the probe on the tissue can affect the measured dielectric properties [35]; therefore, we tried to always apply the same pressure on the samples. We also verified that the pressure applied was the least pressure possible, while ensuring full contact between the tip of the probe and the measured tissue, as well as avoiding air gaps between the two.

We took the Minimum Information for Dielectric Measurements of Biological Tissues (MINDER), proposed in Reference [36], as a guideline to collect experimental data and associated metadata. MINDER specifications indicate a systematic collection of metadata along with dielectric measurement data, in order to support a more informed sharing and re-use of dielectric data. Since measurement confounders (e.g., calibration procedure; calibration drift; validation procedure) and clinical confounders (e.g., tissue origin; animal age and weight; time between excision and measurement; tissue handling procedures; tissue temperature) can impact the measured data [31,37,38], reporting confounder-related metadata supports consistent, interpretable, and accurate dielectric data. The measured data and the related metadata will be made available in the MINDER open-access online repository [39].

#### 2.1.1. Characterization of Human Axillary Lymph Nodes

We measured a total of 11 ALNs from 9 patients diagnosed with breast cancer, which were excised by a trained surgeon during SLNB surgeries scheduled at the University Hospital Galway (Galway, Ireland) [40]. After each ALN excision, the surgeons placed the sample in a container and brought it for dielectric property measurement. The time after excision was at most 20 min, and the sample was measured immediately after receiving it. The average (±standard deviation) temperature of the sample surface (measured at the same moment of the dielectric measurement) was 20.9 °C (±2.3 °C). The size of ALNs ranged approximately from 5 mm to 2 cm on their longer axis, whereas their thickness varied approximately between 4 mm and 7 mm. These thickness values are greater than the sensing depth of the probe which has been estimated to vary between 2 and 3 mm, as reported in Reference [41,42]. The excised ALNs were embedded in fat tissue which was removed by the surgeons as much as possible. However, we note that sometimes fat could not be completely removed, due to the risk of cutting or puncturing the ALN, which would compromise the subsequent histopathological examination. However, the surgeons indicated the best measurement point (i.e., the point on the ALN surface that contained the least fat). The samples were then given to the pathologist for standard histopathological analysis. All the 11 ALNs were diagnosed as “negative”, i.e., no tumor cells were found during the histopathological examination. We do recognize this as the main shortcoming of the present study, and we plan to extend it so that we can gather information from metastasized ALNs. Yet, for the purpose of this paper, we believe the measurements provided here are sufficient for the purpose of phantom development. For each of the 11 ALNs, we measured between 3 to 5 points (depending on the size of the ALN) in which the probe was put in contact with the outer surface of the ALN. In total, we collected 45 measurements from all ALNs.

**Figure 2:**
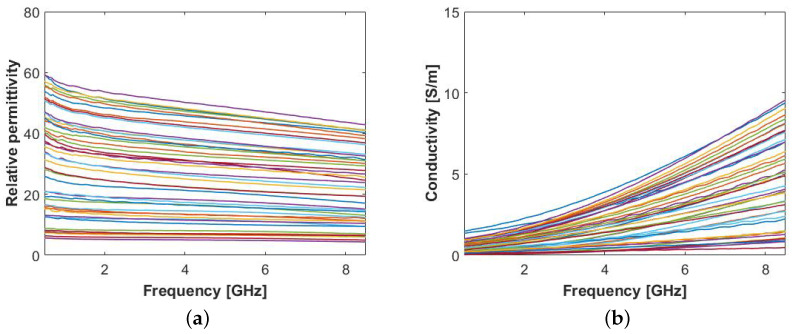
Permittivity results obtained from 11 human axillary lymph node samples measured from the surface layer: (**a**) Relative permittivity, $\epsilon^{\prime }$, and (**b**) conductivity, σ.

The de-embedded dielectric properties, resulting from human ALNs measurements, are illustrated in Figure 2.

The results show very high variability of measured ALN dielectric properties, as the relative permittivity ranges from 5.7 to 50.1 and the conductivity from 0.3 S/m to 4.1 S/m at 4 GHz. These results are in line with those reported by Cameron et al. [28], confirming that the presence of fat on the outer surface of the sample may have a major influence on the permittivity. Due to its high variability, we cannot make definite conclusions about the permittivity of the content of ALNs. However, in order to allow the interpretation of our results, we separated the data into three different groups, defined as follows:

- $Group_{1-10}$ included measurement where $\epsilon^{\prime }\left(4GHz\right)\leq 10$;
- $Group_{10-40}$ included measurement where $10<\epsilon^{\prime }\left(4GHz\right)\leq 40$;
- $Group_{40+}$ included measurement where $\epsilon^{\prime }\left(4GHz\right)>40$.

The lowest values of relative permittivity (i.e., $\epsilon^{\prime }<10$) are a consequence of the fat content on the surface of the ALNs and are not of interest for this study. From the remaining values, we may conclude that ALNs do have higher permittivity than the fatty content that embeds them, indicating that their detection is possible at microwave frequencies. Furthermore, we note that the ALNs in which the surgeon was able to remove the largest amount of fat from the surface correspond to the highest permittivity values ($\epsilon^{\prime }>40$ at 4 GHz) and show high consistency across intra-sample measurements. Considering that consistency is a valid indicator of tissue homogeneity, we presumed that $Group_{40+}$ dielectric values effectively correspond to the dielectric properties of the ALN under test.

Lastly, for $Group_{10-40}$ and $Group_{40+}$ ($Group_{1-10}$ was excluded due to the above mentioned reasons), we fit the mean permittivity to a two-pole Debye model using the Least Squares Method (LSM) [43] to minimize the fitting error, which is a widely adopted approach to retrieve the 2-pole Debye model parameters [44]. The parameters are reported in Table 2. The fitting error, defined as the mean (over frequency) absolute difference between measured data and model, was at maximum 0.38 regarding relative permittivity and 0.11 S/m regarding conductivity, demonstrating that the model is a good representation of the measured data.

#### 2.1.2. Characterization of Animal Lymph Nodes

As explained in the previous sub-section, the fat surrounding human ALNs affects the permittivity measurements and allows only limited conclusions to be drawn. Ex-vivo animal tissues do not have the handling constraints, such as with human tissues. This allowed us to get data from their inner cross-section and measure their permittivity more accurately.

For the purpose of this study, we measured eight LNs from two sheep corpses. The animals were healthy ewes of mass approximately 70 kg and about 6 years old. The LNs were excised by a trained veterinary surgeon from the inguinal area, approximately 3 to 4 h after the death of the animal, and placed in a closed container. No preservatives or additives were used as these could impact the dielectric measurements. The surgeon removed as much fat as possible with a scalpel, avoiding puncturing the LN capsule (i.e., a thin layer of connective tissue covering the node [45]). We note that, since in this case we had no handling nor ethical constrains, we were able to remove more fat from animal LNs compared to human ALNs. We performed the measurements within 20 min to 4 h after tissue excision.

**Figure 3:**
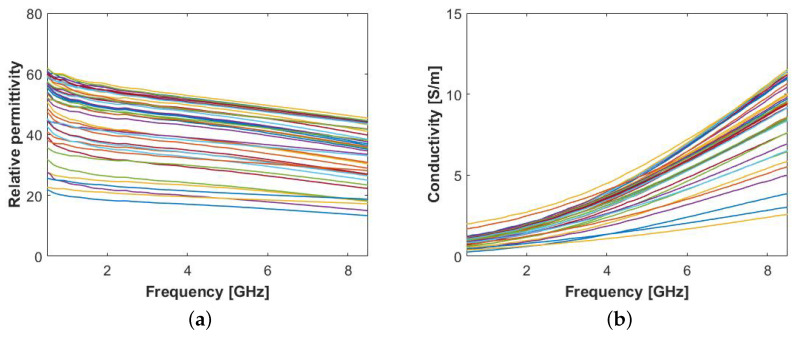
Permittivity results obtained from animal lymph node surface layer: (**a**) Relative permittivity, $\epsilon^{\prime }$, and (**b**) conductivity, σ.

We first measured the LNs on the outer surface for a direct comparison with human ALNs. The eight samples were measured in 4 to 6 locations depending on the size of the LN, in a total of 41 measurements, while trying to avoid measurements near regions with residual fat. The average (± standard deviation) temperature of the LN surface was 20.6 °C (±1.3 °C). Figure 3 illustrates the de-embedded permittivity. Similarly to the permittivity results plotted in Figure 2, there is a significant variability between measurements, due to the presence of residual fat. Nevertheless, we can observe that both the maximum permittivity values ($\epsilon^{\prime }$ around 50 at 4 GHz) and the dispersive behavior (σ in the range of approximately 1 S/m to 4 S/m at 4 GHz) are similar to those in Figure 2.

**Figure 4:**
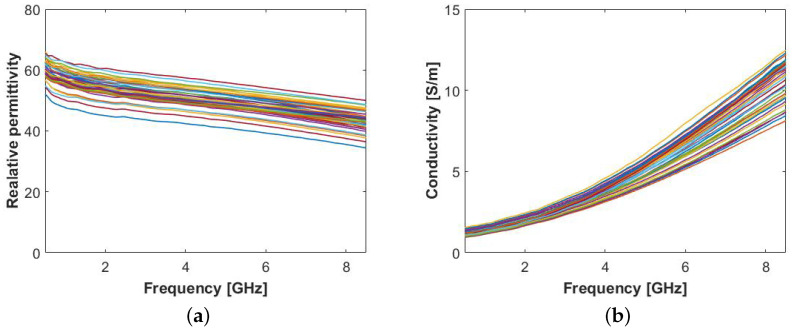
Permittivity results obtained from animal lymph node inner layer: (**a**) Relative permittivity, $\epsilon^{\prime }$, and (**b**) conductivity, σ.

For each LN, immediately after the outer surface measurement, we sliced the sample in half and placed the probe in contact with the LN cross-section surface. Again, we took measurements in 4 to 6 locations for each LN, in a total of 40 measurements. The average (±standard deviation) temperature of the sample surface was 21.8 °C (±0.9 °C). Figure 4 shows the permittivity obtained from the core of the animal LNs.

In contrast with the permittivity obtained measuring the LN surface, measurements on the inner cross-section are consistent with one another due to the homogeneity of the interior of LNs. The relative permittivity and conductivity of LNs at 4 GHz is about 55 and 4 S/m, respectively. These values are quite similar to the highest permittivity values obtained from the surface measurements of both human ALNs and sheep LNs (see Figure 2 and Figure 3, respectively). Such high permittivity values are indicative of very high water content, which is compatible with the physiological function of the organ.

Lastly, we fit the mean permittivity of all the cross-section measurements to a two-pole Debye model, following the same procedure described in Section 2.1.1. The Debye models’ parameters we obtained are reported in Table 2. The absolute fitting error was at maximum 0.30 units in relative permittivity and 0.05 S/m in conductivity, demonstrating that the model is a good representation of the measured data.

## 3. Tissue Mimicking Materials

In this section, we produced the TMMs that represent the dielectric properties of each tissue (fat, muscle, bone, ALN, and lung) at microwave frequencies.

**Figure 5:**
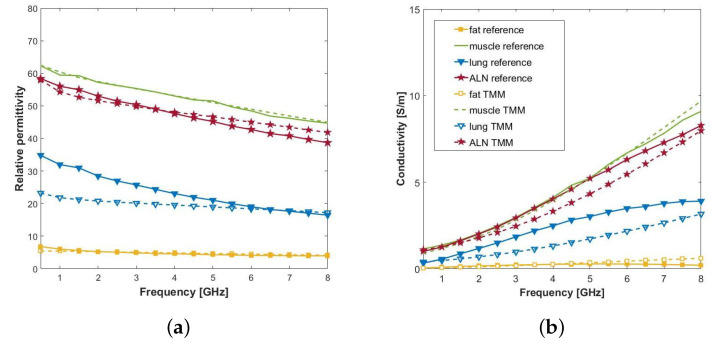
**Solid lines**: reference permittivity of the four tissues of interest: fat, muscle, lung, and axillary lymph nodes (ALN). **Dashed lines:** measured permittivity of the corresponding liquid tissue mimicking materials (TMMs). (**a**) Relative permittivity, $\epsilon^{\prime }$, and (**b**) conductivity σ.

We defined the reference dielectric properties for fat, bone, and lung tissue referring to the data published in Reference [29] (available on the IT’IS Foundation database [46]). Specifically, using the same terminology as [29], we referred to “breast fat”, “cancellous bone”, and “inflated lung”. We instead used data retrieved from measurements performed on ex-vivo bovine muscle tissue [30] to assign the muscle dielectric properties. As for ALNs, we referred to the permittivity measurement described in Section 2. In particular, we considered the measurements performed on the surface of a “non-fatty” human ALN, which showed high consistency over intra-sample dielectric measurements. The reference dielectric properties of fat, lung, muscle, and ALN tissues are reported in Figure 5.

With the exception of bone, we represented all the tissues with liquid TMMs, as they are easy to produce and stable over time, when preserved in a closed container. The bone structure was 3D-printed with a solid conductive polymer Conductive PLA 2.85 mm 500 g from Protoplant, Inc., Vancouver, WA, USA [47], because of its intricate shape. This polymer, known as “Protopasta Conductive PLA” presents dielectric properties similar to those of the cancellous bone tissue in our frequency band [48].

The liquid TMMs are mixtures composed of different amounts of Triton X-100 (Triton X-100 from Merk KGAa, Darmstadt, Germany [49])—which will be referred as TX-100, water, and sodium chloride (NaCl), according to the recipe described in Reference [23]. The column on the left in Table 3 reports the quantities of TX-100 and NaCl for each tissue, while Figure 5 shows the corresponding measured permittivity using the OECP technique.

The average (±standard deviation) absolute errors in terms of relative permittivity and conductivity (1 GHz to 8 GHz frequency band), are reported for all tissues in Table 3. There is good agreement between fat, ALN, and muscle TMMs and reference permittivities with a mean square error lower than 6.7% in the 1 GHz to 8 GHz frequency band. Regarding lung, the TMM permittivity is 50% to 14% below the adopted reference value, for frequencies between 0.5 GHz and 4.5 GHz. However, lungs are at a greater depth than the muscle, which means that it is the tissue that has the least influence on the E-field coupling in the ALN region, and so the model mismatch will not affect the work presented in this paper (we will show this in Section 5). In addition, as discussed in Reference [29], it is estimated that the dielectric properties vary by as much as 10% between individuals at microwave frequencies. Therefore, the proposed liquid TMM is still a viable mixture to mimic the lungs for the proposed application.

## 4. Axillary Phantom Design and Development

In this section, we address the development, and fabrication of the 3D-printed phantom. Section 4.1 briefly describes the envisioned ALN MWI device that is under development, which justifies the choice of the patient position adopted for our model and the choice of the tissues to include in the phantom. Section 4.2 addresses the segmentation of the CT scan and the development of the numerical model of the axillary region. Additionally, it presents the 3D-printed phantom that we fabricated.

### 4.1. Patient Position and Intended MWI Setup Description

**Figure 6:**
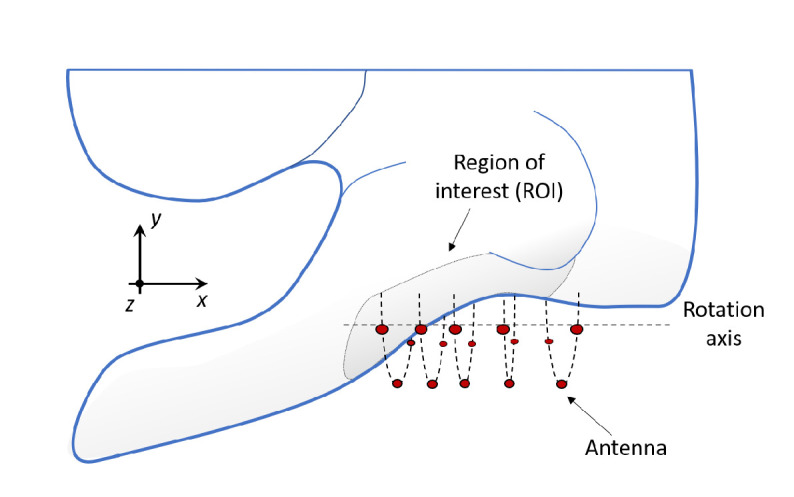
Illustration of the setup for axillary lymph node microwave imaging. The patient lays in a supine position, while the antennas scan the underarm region.

In order to enable access to the axillary region for imaging, the patient should be laying in a supine position with the arm extended along the head, as sketched in Figure 6. The antennas rotate around the underarm, thus illuminating the ALNs and collecting their microwave response from multiple perspectives. The proposed positioning of the patient has significant impact on the phantom development, and we took this into consideration when choosing the reference CT scan to build our phantom.

In contrast with most medical MWI setups, in which the part of the body being scanned is immersed in a liquid [17,50,51], the envisioned setup is dry and fully contactless. Two main reasons motivated this choice: the first is that the shape of the underarm hinders its immersion and the usability of the setup; the second is that the liquid may raise concerns about hygiene between examinations. Some groups working on medical MWI have demonstrated the feasibility of such a dry approach [52,53], including some authors of this paper.

### 4.2. Axillary Phantom Development and Fabrication

We developed our phantom based on a thoracic CT, of a woman who had been diagnosed with breast cancer and was undergoing treatment at the Champalimaud Foundation [54] (Lisbon, Portugal). The patient was 68 years old, 154 cm tall, and weighed 50 Kg (body mass index BM = 21.1).

During the acquisition, the patient was in a supine position, with her arms extended along the head, which is the standard position for CT breast cancer imaging. Note that such posture meets our model requirements, as explained in Section 4.1. CT images provide high and uniform contrast in all the body region, thus enabling accurate segmentation of the tissues.

**Figure 7:**
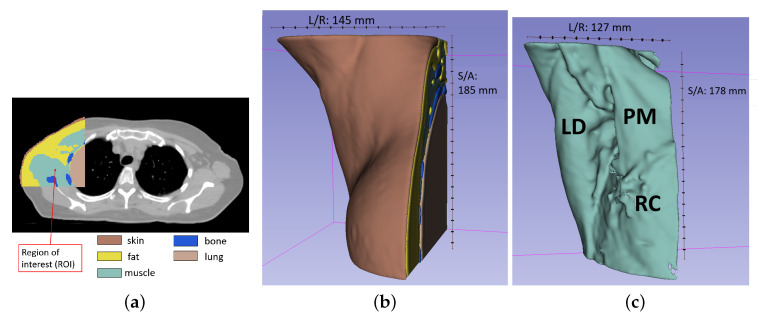
(**a**) Axial view of the body at the level of the sternum: the segmented organs are superimposed to the original Computed Tomography (CT). Each tissue is represented by a different color (legend). The red arrow points at the segmented region of interest. (**b**) 3D-rendering view in 3D-slicer of the segmented organs. (**c**) 3D-rendering view in 3D-slicer of the segmented muscle tissue. Different muscles of the region are indicated: pectoralis major (PM), latissimum dorsi (LD), and rib-cage (RC) muscle. Superior/Anterior (S/A) and Left/Right (L/R) dimensions of the model are reported.

The CT consists of several transversal planes (corresponding to the xy-planes if referring to the system in Figure 6). The overall resolution of the scan is 1.17 × 1.17 × 2 mm^3^ in a total of 512 × 512 × 130 voxels. We limited the size of the Region Of Interest (ROI) to a volume of 145 × 145 × 180 mm^3^, in order to fit it into our 3D-printer (Form3, Formlabs [55]). We note that the selection of such a restricted volume does not compromise the application, because the ALN region (i.e., the sub-region where the ALNs are located) is limited to the fatty region of the underarm. Figure 7a shows one axial plane of the CT scan, where we have highlighted the segmented ROI. Each color within the ROI represents a different tissue. We identified five tissues of interest, which were segmented from the CT scan: fat, muscles, bones, lung, and skin. We performed the segmentation with the aid of 3D Slicer 4.10.2, a free open source software platform for biomedical imaging research [56,57], which is widely used in medical additive manufacturing. In order to segment the tissues of interest, we applied a threshold on the voxel Hounsfield unit (HU), which is the most widely used segmentation method in medical additive manufacturing [58]. For each tissue, we manually chose the optimal threshold values (upper and lower HU bounds) to allow an accurate segmentation of each organ. It should be noticed that, as reported in Reference [59], the use of manual threshold selection, though subjective, has been proved to be a reliable method for accurate segmentation.

The choice of the threshold is relevant, in order to prevent non-continuous surfaces (i.e., discontinuities) or structures that are split into separate volumes, which have to be solved ahead of 3D-printing. After thresholding, we applied morphological closing filtering (with a 3 × 3 × 3 voxel cubic structuring-element) to each segment to fill small holes that resulted from the segmentation while preserving the shape of the segments. We then dealt with these by manually closing the discontinuities and merging the separate volumes of the same tissue. For example, the pulmonary bronchi and alveoli were segmented together with the region of the lung tissue, even though their HU did not originally correspond to the adopted lung HU range. Figure 7b shows the final 3D-rendering of the segmented volume. In addition, Figure 7c illustrates the segmented muscle, after we manually closed the surface discontinuities and merge some separate volumes, in order to be able to 3D-print it as one piece.

The numerical model obtained from the CT presented intricate morphological details due to the intrinsic complexity of the anatomical structures. Given that we aimed to produce a phantom compatible with 3D-printing technology, we reduced the complexity of some details of the model, in order to reduce the amount of polymer of the fabricated phantom, thus mitigating the possible influence of polymer in microwave measurements [60]. We ensured that the main features of the phantom were still present in the final phantom, and only small details were reduced or removed. Firstly, we merged the four main muscles (pectoralis minor, pectoralis major, latissimus dorsi muscle, and rib-cage muscles, represented in Figure 7c) in a single structure, due to their proximity and significant reduction of the complexity of the overall structure. Secondly, we removed the rib-cage bones, since they are embedded in the pectoralis and rib-cage muscles. We considered that adding bones would significantly increase the complexity of the phantom, while their effect on the microwave signals would be negligible, due to the high dielectric properties of the surrounding muscles. Additionally, we smoothed the surface of the segmented tissues in Meshlab [61], in order to mitigate some irregular or sharp edges that resulted from the segmentation. Laplacian smoothing [62] was first applied to the entire organs and then locally to regions which presented particularly rougher surfaces.

The final step in the creation of the phantom was the conversion of the muscle and lung solid structures to closed surface ones (i.e., hollow structures). On these two surfaces, we included an aperture, in order to enable filling them with the appropriate liquid TMM. Moreover, we added three slabs to the skin model, obtaining a partially-closed hollow structure which can be filled with the fat liquid TMM. Both these steps were performed with software Blender [63].

**Figure 8:**
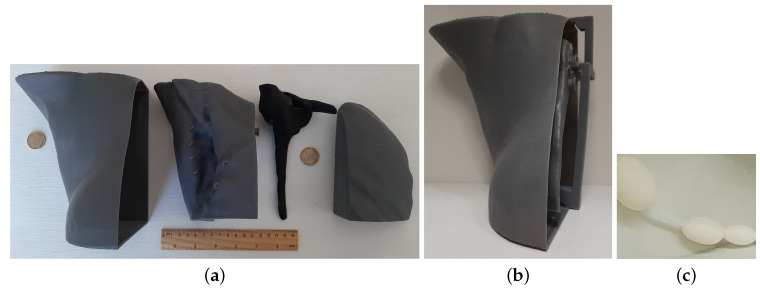
Three-dimensional-printed phantom. (**a**) From left to right: (i) torso, (ii) muscle, (iii) bone, and (iv) lung cavities, together with the iii) bone structure are represented. (**b**) Assembled phantom, where we can observe an extra part which keeps the organs in place inside the other container. (**c**) Three ellipsoidal hollow axillary lymph nodes, with a wire passing through them.

We 3D-printed the lung, muscle and fat containers with 3D-printer Form 3 (Formlabs [55]) using the Grey Resin polymer (Formlabs [55]) and a wall-thickness setting of 1.2 mm. The bone was printed as a solid part using the Protopasta Conductive PLA [47] with 3D-printer Ultimaker 3 [64] using a layer height of 0.2 mm. Figure 8a shows the 3D-printed organs considered in the phantom. Figure 8b illustrates the assembled phantom. In the same figure, it is possible to observe an extra holding part which ensures the organs are static and in a consistent position. Moreover, note that the outer container (i.e., fat container) is open on one side, in order to have easy access to the inside of the phantom. Only the muscle and lung containers include an aperture to allow filling them with TMMs.

Regarding the ALNs, we created a set of hollow ellipsoids with sizes ranging between 5 mm to 20 mm on their longer axis, thus approximating the anatomical shape and size of ALN [45]. The modelled ALNs include an aperture on both extremities, in order to enable filling them with the appropriate liquid. Additionally, the ALNs may be organized in a small network, by connecting a flexible plastic tube between them. A set of ALNs, represented in Figure 8c, was 3D-printed in STRATASYS F170 [65] using acrylonitrile butadiene styrene (ABS). With this strategy, we may place a single or a network of ALNs inside the phantom, thus increasing its representativeness of the axillary region. We would like to note that the ALN network may be placed in different positions, allowing for variability of the overall phantom. The ALNs are attached to the muscle structure through a set of small rings using a nylon string (notice the surface of the muscle container in Figure 8a).

## 5. Numerical Assessment

The proposed phantom is composed of multiple polymeric containers which hold the TMMs. However, it is well-known that polymers have a relative permittivity between 2 and 3, which is lower than the permittivity of most tissues. Therefore, this section aims to quantify the differences between our phantom and the real axillary region through full-wave simulations. All the numerical results presented below were obtained using Computer Simulation Technology (CST) Microwave transient solver [66].

**Figure 9:**
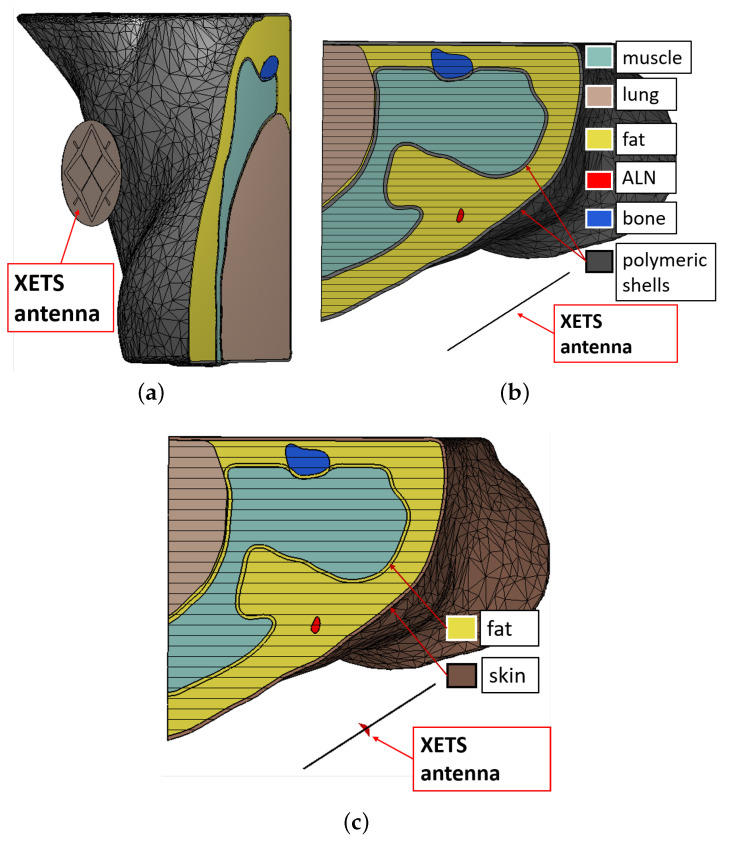
(**a**,**b**) Numerical model of the developed physical phantom (*Phantom*). (**a**) 3D-rendering view; (**b**) axial view at the level of the sternum. (**c**) Numerical model of the realistic axillary region (*Axilla*), in axial view. Each color represents a different component (legend). A crossed Exponentially Tapered Slot (XETS) antenna is placed close to the torso. Model size: 185 mm × 145 mm × 145 mm.

In order to assess the influence of the 3D-printed polymeric containers, we considered two setups. The first setup (*Phantom*) included the polymeric walls of the phantom, with a thickness of 1.2 mm. We assigned a dielectric constant of 2.5 and a loss tangent of 0.02 to represent the polymer, which was measured using the microstrip method proposed in Reference [67]. Figure 9a (cross-sectional view in Figure 9b) illustrates the model. The grey surfaces represent the polymer. The second setup (*Axilla*) represented the realistic axillary region, which does not include any polymeric wall adopted for the phantom, while it includes a skin layer of 1.2 mm thickness. The latter is shown in Figure 9c. Note that there are no grey surfaces, and only an external brownish layer mimicking the skin is visible. In both setups, the tissues were assigned the reference complex permittivity values plotted in Figure 5. We referred to the literature [29] to assign the skin tissue properties.

**Figure 10:**
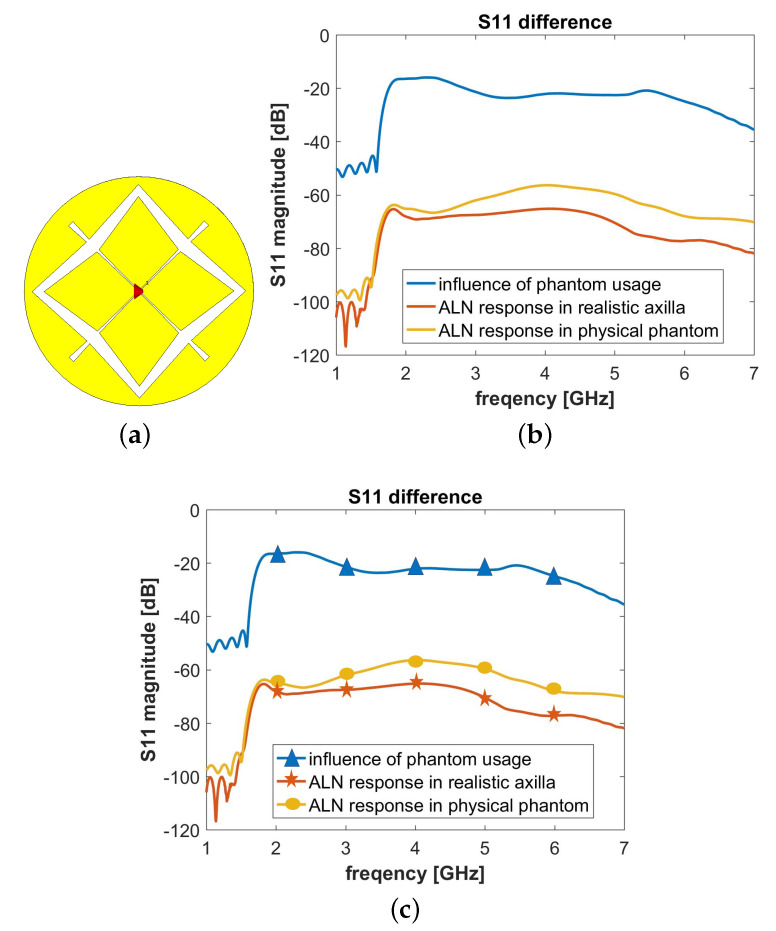
(**a**) Schematic of the crossed Exponentially Tapered Slot (XETS) antenna. (**b**),(**c**) Influence of the usage of the developed phantom on the reflection coefficient ($S_{11}$). (**b**) Comparison between the $S_{11}$ magnitudes measured by the XETS when stimulating the *realistic axillary region* and the *developed physical phantom*; the $S_{11}$ of the antenna in free-space is also reported. (**c**) Magnitudes of the difference between the $S_{11}$ measured in three different pair of cases (see legend).

As for the electromagnetic source, we used a broadband antenna operating in the 2 GHz–6 GHz frequency band which is represented in Figure 10a. The antenna is composed of a crossed Exponentially Tapered Slot (XETS) and was previously reported in Reference [68,69]. The latter study [69] includes the dimensions of the antenna used in the present study. The antenna is well suited for imaging applications not only for its impedance matching over a broad bandwidth but also because it presents a stable phase centre along the frequency. Figure 10b represents the magnitude of the input reflection coefficient, $|S_{11}|$, of the antenna in free-space, which is below −10 dB, as is common practice in antenna design. The position of the antenna in the two setups is visible in Figure 9.

**Figure 11:**
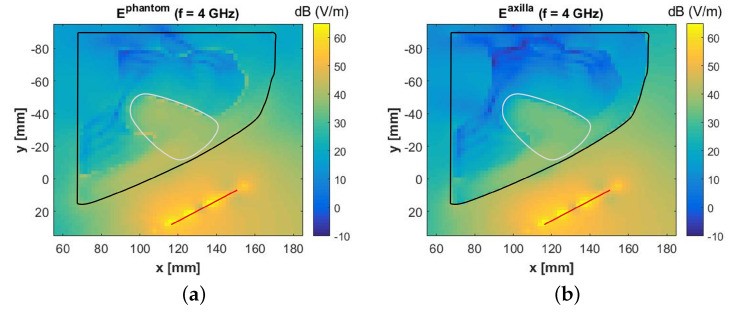
Magnitude of the electric fields (f=4 GHz) computed, at the level of the sternum, when considering two different setups. (**a**) $E^{phantom}$, referring to the setup in Figure 9b. (**b**) $E^{axilla}$, referring to the setup in Figure 9c. The electromagnetic source is the crossed Exponentially Tapered Slot (XETS) antenna illustrated in Figure 10a. The black line identifies the contour of the axillary region (i.e., polymeric external shell in case (**a**) and skin in case (**b**)). The white line identifies the axillary lymph node region. The red line identifies the antenna.

We computed the E-field considering the two setups. The magnitude of the E-fields, $|E^{phantom}\left(f\right)|$ and $|E^{axilla}\left(f\right)|$, at f=4 GHz are plotted in Figure 11a,b, respectively. We observe that the E-field has a magnitude of about 30 V/m to 40 V/m in the ALN region in both scenarios, thus suggesting the polymer has little influence on the results. Moreover, the E-field that couples to the muscle is around 20 dB below the E-field in the region of the ALN (circled in Figure 11). This is a consequence of the losses and of the high dielectric contrast between muscle and fat, which causes a very large reflection on the interface between the two tissues. As a result, we conclude that having limited the volume of the phantom to 145 × 145 × 180 mm^3^ to fit in our printer minimally impacts the measurements.

In order to better understand the influence of the polymer, we calculated the difference of the E-field as

(3) $$E_{dB}^{diff}=20log_{10}|E^{axilla}|-20log_{10}\left|E^{phantom}\right|.$$

**Figure 12:**
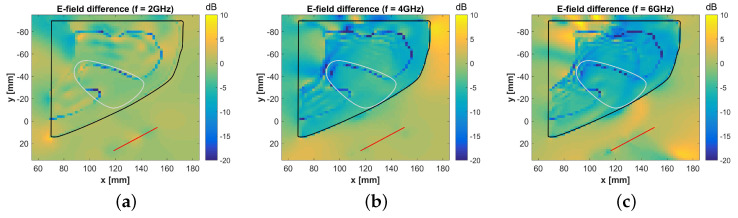
Effect of the developed phantom on the energy coupling inside the axillary region tissues. The plot represents the difference between the total field computed in *Axilla* model ($E^{axilla}$) and the total field computed in the *Phantom* model ($E^{phantom}$). The electromagnetic source is the XETS antenna illustrated in Figure 9a. The black line identifies the contour of the axillary region (i.e., skin or phantom surface in cross-sectional view). The white line identifies the axillary lymph node region. The red line identifies the antenna. (**a**) Frequency f=2 GHz; (**b**) f=4 GHz; (**c**) f=6 GHz.

Such E-field difference is plotted in Figure 12 at 2 GHz, 4 GHz, and 6 GHz. The results show that $E_{dB}^{diff}$ varies between −2 dB at 2 GHz and −8 dB at 6 GHz, in the volume corresponding to the ALN region. Considering the advantages brought by the use of a 3D-printed phantom, we consider that the reported E-field differences are acceptable.

We extended the present analysis to the assessment of the impact of the polymer on the input reflection coefficient of the antenna, $S_{11}$. To this end, we calculated the difference on the $S_{11}$ between the two numerical setups represented in Figure 9 as

(4) $$S_{11}^{diff}=20log_{10}\left(\left|S_{11}^{phantom}-S_{11}^{axilla}\right|\right),$$

where $S_{11}^{phantom}$ and $S_{11}^{axilla}$ represent the simulated $S_{11}$ with the phantom and the realistic axillary region, respectively. The results are plotted in Figure 10c. We observe that the influence of the polymer is lower than −20 dB, thus proving that the influence of the polymer is not significant.

Lastly, we analyzed the response of the ALN along the frequency when embedded in the phantom, $S_{11}^{ALNph}$, and in the realistic axillary region, $S_{11}^{ALNax}$. We included a kidney-shaped ALN of 7 mm length on its longer axis (a representation is reported in Figure 9) in the numerical setups and a permittivity assigned as in Section 3. We inferred the response of the ALNs by calculating

(5) $$S_{11}^{ALNph}=20log_{10}\left(\left|S_{11}^{phantom}-S_{11}^{phantom+ALN}\right|\right),$$

(6) $$S_{11}^{ALNax}=20log_{10}\left(\left|S_{11}^{axilla}-S_{11}^{axilla+ALN}\right|\right),$$

where $S_{11}^{phantom+ALN}$ and $S_{11}^{axilla+ALN}$ are the simulated input reflection coefficients of the phantom with ALN and of the realistic axillary region with ALN, respectively. These results are shown in Figure 10c. The response of the ALN is in the order of −60 dB and −70 dB for the physical phantom and for the axilla, respectively, in the frequency band between 2 GHz and 6 GHz. We may consider that this response is sufficiently high, given that commercial VNAs have a dynamic range of a least 90 dB, which would be sufficient to detect a useful response of the ALNs. In addition, the magnitude of such response is comparable to the one experimentally obtained for breast tumor imaging in Reference [53], where the authors successfully detected the tumor in the correct position.

## 6. Conclusions and Future Work

We developed the first axillary phantom for experimental assessment of MWI for ALN screening. To reach this goal, we improved the literature knowledge on LN dielectric properties, which represents the complementary accomplishment of this work.

We estimated the relative permittivity of healthy ALNs to be in the range of 45 to 60 at 4 GHz. Despite the challenges due to fat surrounding the ALNs, this was possible since consistency across measurements sites is a valid indicator of the homogeneity of the tissue being measured, while low dielectric properties are an indicator that fat is being measured. In addition, measurement of animal LNs strengthened our conclusions.

The developed physical phantom originates from the 3D-printing of a CT segmented series of images; therefore, it is anatomically realistic, and it reproduces fine morphological details. The employment of polymeric containers allowed us to use liquid TMMs, which is a great advantage since they are easy to produce and their dielectric properties were proved to mimic the axillary tissues with good accuracy. As a disadvantage, full-wave simulation showed that that the polymer tends to influence the coupling of the E-field inside the phantom. After testing the developed phantom numerically, we concluded that the usage of our 3D-printed phantom alters the E-field coupling into the tissue of 7 dB at maximum at the central frequency (f= 4 GHz). We also have to report that the physical phantom construction involved a slight simplification of the original anatomically realistic numerical phantom, which was necessary to solve the trade-off between polymer-presence and anatomical-details.

As a last result, we assessed the response of a relatively small ALN inside the axillary region. We estimated such response to be sufficiently large (−60 dB to −70 dB) to be sensed by our VNA, which motivates further studies on ALN MWI.

## Figures and Tables

**Table 1:** Dielectric measurement validation result: mean percentage difference, considering all measurement sessions, between the permittivity inferred from the validation measurements and the reference values. Data are separated between pre-measurement validation (V1) and post-measurement validation (V2). Moreover, the left side of the table refers to human axillary lymph node (ALN) measurements, while the right side of the table refers to animal lymph node (LN) measurements.

	Human ALNs		Animal LNs	
	$\Delta \epsilon^{\prime }$ [%]	Δσ [%]	$\Delta \epsilon^{\prime }$ [%]	Δσ [%]
**V1**	1.06	2.09	0.93	2.93
**V2**	2.26	2.92	5.50	2.13

**Table 2:** Two-pole Debye parameters fitted to the mean permittivity computed on three different groups of data. The first two rows refer to human axillary lymph node data, divided into two different groups, according to their relative permittivity values. The third row refers to all the data acquired when measuring the inner content of sheep lymph nodes.

	$\epsilon_{\infty }$	$\sigma_{s}$ [S/m]	${\Delta \epsilon }_{1}$	$\tau_{1}$ [s]	${\Delta \epsilon }_{2}$	$\tau_{2}$ [s]
ALN $Group_{10-40}$	1.00	0.96	34.17	7.05× 10 ${}^{-10}$	16.22	1.36× 10 ${}^{-11}$
ALN $Group_{40+}$	1.00	1.23	119.39	1.0× 10 ${}^{-9}$	38.25	1.0× 10 ${}^{-11}$
Animal LN (inner content)	0.97	1.44	211.99	n1.65× 10 ${}^{-10}$	45.18	1.12× 10 ${}^{-11}$

**Table 3:** Liquid tissue mimicking material mixture recipe. Muscle, fat, lung, and axillary lymph node liquid TMM are made of a liquid mixture of TX-100 and salted water. The first column of the table reports, for each liquid TMM, the percentage volume of TX-100 and the concentration of NaCl in deionized water. The second column of the table reports the average (± standard deviation) absolute error, in terms of permittivity and conductivity, computed for each TMM properties with the respect to the corresponding target properties.

Tissue	Mixture Composition		Average (±st.dev) Absolute Error in TMM Dielectric Properties	
	TX-100	NaCl	Relative Permittivity	Conductivity
	[vol%]	[g/l]		[S/m]
muscle	26.5	7.4	0.4 (±0.3)	0.21 (±0.13)
lung	55.0	4.51	4.2 (±3.7)	0.89 (±0.43)
fat	100	0	0.3 (±0.2)	0.11 (±0.11)
ALN	25.0	8.59	1.6 (±0.9)	0.33 (±0.21)
